# On the Mental Manifestations and Impulses of the Insane
*This essay is from the pen of an accomplished and intelligent gentleman, but not in the profession. His remarks are based upon his own observation and study of a numerous class of mental cases which have been under his own care.—Ed.


**Published:** 1851-07-01

**Authors:** 


### ON THE MENTAL MANIFESTATIONS AND IMPULSES OF THE INSANE.*

As instruments of the finest tone are most easily injured by a rude or unskilful touch, the most gentle and refined are most liable to suffer from whatever tends to injure the nervous system. Whatever the previous character of the sufferer, it cannot be too strongly impressed on the minds of junior members of the medical profession, as well as on the friends and attendants of the insane, that, in their incipient stages, no certain prognosis can be arrived at of what are usually termed mental diseases; for acute mania, tending to suicidal, homicidal, and other melancholy results, may be preceded by fantastic tricks and absurd vagaries, which seem only to denote the most harmless folly, or by a meek and pensive gentleness which touches all hearts, and lulls every suspicion of dangerous impulses. The few following exemplifications of these general statements, we trust, may not prove unworthy the perusal of such students of mental pathology as have not yet had the advantage of protracted and diversified experience.

Case 1.—A schoolfellow of the writer of this article, a youth of excellent moral conduct and good intellect, and of very gentle manners, had become deranged while serving as a subaltern officer in the West Indies. His freaks, however, were those of a mere schoolboy—*riding a great gun* being, indeed, his favourite pastime. Morning after morning, attended by the soldier who acted as his keeper, he proceeded to a favourite battery, mounted his *mettled steed,* and went through all the actions and attitudes of a warrior bestriding his charger in full career,—greatly to his own satisfaction, and to the unavoidable amusement, melancholy as mental alienation must ever be in reality, of the by-standers. At last, as his recovery seemed hopeless, a medical board recommended his being sent to England, and placed on half-pay. The day on which the vessel, on board which his passage had been engaged, was to sail, he was led unresistingly to the landing-place, and entered the boat awaiting him without reluctance; but when about half-way between the shore and the ship, affected probably by the motion of the waves, he sprang to his feet—no precautions against violence having been thought needful—bit, struck, and kicked furiously; and was only secured with the utmost difficulty, after exposing himself and the seamen, and others who accompanied him, not only to mechanical injuries, but to the danger of a watery grave, as much skill was required to keep the boat *trimmed,* until he was overpowered. We believe that after this sally he became again inoffensive, and gave no further trouble.

Case 2.—In the year 1834, Mons. Louis Costil, a French protestant, of middle age, unexceptionable morals, and professing a steadfast belief in religion, while residing at T—, as a teacher of his native language, and

* This essay is from the pen of an accomplished and intelligent gentleman, but not in the profession. His remarks are based upon his own observation and study of a numerous class of mental cases which have been under his own care.—Ed.

portrait and miniature painter, had a severe bilious fever, preceded by obstinate congestion of the liver. It may be well to observe here, that long before his indisposition was manifested, he had often been heard groaning in his bed-room at night, although exhibiting all the hilarity of his nation by day. Some years before, after a similar but less grave attack, he had appeared "rather odd" for a few weeks; and, on this occasion, when his general health seemed restored, it was but too evident that his intellect was seriously affected. Hitherto most frugal, and punctual in all his pecuniary transactions, he now ran in debt without attempting to pay any one; and, when expostulated with on the subject, only laughed, and seemed to think his conduct a good joke. He cut and defaced paintings and drawings, the sale of which would have amply sufficed to discharge his very limited liabilities; abandoned all professional occupations; went to meals at houses unasked—and sometimes where he was not personally acquainted; and stole flowers from gentlemen's gardens—a feat which seemed to afford him the greatest delight. His chief hobby, however, and which he called his *Lettro-mania,* was addressing letters alike to friends and strangers—some replete with good sense and piety—some frivolous and absurd, and some a compound of sense and nonsense. The gentry of the place and parochial authorities made every effort to provide him with decent lodgings and the necessaries of life, while awaiting the instructions of his friends, who resided in the South of France. Meanwhile, he appeared so thoroughly the happy "madman gay," discoursing most eloquently, among other subjects, on the beauty and perfections of an imaginary fair one in his native land, his love-strains most ridiculously contrasting with his years and appearance, that no precautions were adopted to place him under superintendence. A retired military officer, however, who had seen a good deal of mental fluctuations in derangement, while leading the wandering life of a soldier, predicted to some of the old inhabitants a serious termination of his malady; and, unfortunately, the prediction was but too true; for, not long after, poor M. Costil was found one morning in his bed-room with his throat cut, and quite lifeless.

Case 3.—As in the tropics the *white squall* suddenly interrupts the profound calm, heralded merely by a mere speck of vapour in the bright still sky, observed only by the experienced mariner, the outburst of dangerous mania may occur in the midst of treacherous tranquillity.

Miss A. F—, a young lady of great personal attractions, with a highly-cultivated intellect, refined taste, and of a devotional character, aged about twenty-three, in easy circumstances, and residing with a very sober-minded, elder, unmarried sister, in a manufacturing town in the north of England, had occasionally, previously to and during the earlier part of the year 1828, shown slight symptoms of derangement; but was at all times so gentle and docile, that when her *thoughts wandered a little,* she was merely kept within doors for a few days, until the attack passed off; her state being carefully concealed from all but her nearest relations. Sometime towards the midsummer of the above year, she left home one morning, apparently quite well, to collect for a religious society; but her manner appeared flighty at some houses, where she called on this errand. After completing her rounds, she was seen to leave the town, as if for a country walk—alas, to be brought back, ere long, a corpse!

In less than an hour after her departure from one of the city gates, a child residing at a village two miles distant, ran from the road to the house of its parents in great terror, screaming something about a lady in white who had walked into the pond,—a contiguous pool of filthy stagnant water. At first, no notice was taken of the little fellow's alarm; but on several other children following wildly at full speed, all telling the same tale, some of the villagers hastened to the spot with such implements for dragging as they could find at hand, and soon discovered the body, still warm; but, from the nature of the symptoms, it was apparent that asphyxia had probably taken place instantaneously. The narrator of this sad tale, who had often met the young lady in society a few years before, had, on many occasions, from the peculiar ethereal expression of her pale, intellectual countenance, and sudden scintillations of her bright blue eye, while seated at the piano, thought how highly Raphael would have prized such a model for a St. Cecilia. Females of this temperament are not, however, exempted from the morbid emotions of their sex—perhaps much more liable to them than such ladies as Goldsmith's "Big Bet Bouncer,"* Dr. Lever's "Baby Blake,"† or Crabbe's "Widow Go."

> "A sturdy dame,
> Famed ten miles round, and worthy all her fame."

We well know that highly moral and intellectual *men*, in whom the nervous system generally predominates, and whose intellectual labours, while they overtask the brain, leave the muscular system unexercised, are often absolutely tormented by blasphemous and unclean thoughts in periods of bodily exhaustion, or when suffering from visceral diseases. Such men may be of any religious denomination, Roman Catholics, or Neologists, High Churchmen, Low Churchmen, or Deists aspiring to human perfection. The amiable Pascal was often but too happy to have recourse to what philosophy would consider *ecclesiastical play-things*, and take a part in decorating altars, and arranging ornaments in churches, to divert his mind. How terrible, then, must unholy thoughts, however involuntary, be to females of mental refinement and the religious idiosyncrasy. "My heart is like a nest of unclean birds," was the melancholy expression of a most exemplary widow lady, under middle age, of the bilious temperament, who once requested our opinion on the expediency of entering an asylum. The sudden death of her father, although a man above fourscore, rendered this painful step necessary soon afterwards—from the intense grief it superadded to her other sufferings. Domenicho has depicted St. Catherine tempted by two brutal, sensual-looking monsters in human form, one of whom lies vanquished, while the other continues his obscene importunities; the holy maiden looking upward with supplicating anguish to a chubby cherub, with a very rotund abdomen, coming to her aid, and whose temperament is certainly not that of an angel likely to be shocked by trifles. The impure suitors seem much more likely to inspire disgust than aught else; but perhaps the painter meant them as types of unholy thoughts visiting the holy. This devotee is a rather *chlorotic-looking* maiden, pallid, and obviously liable to undue excitement of the nervous system.

Byron, to a great extent insane himself, says, in his "Manfred," describing a beautiful maniac—

> "her thoughts
> Had wandered from their dwelling; and her eyes—
> They had the look that is not of the earth."

When this unearthly look is observed in young people of either sex, let parents, friends, and teachers beware. The transition from hyperæsthesia to dysæsthesia is but too common an occurrence, although too little considered for any practical purpose in hygienic education. To revert for one moment to Miss A. F——, there seems to have been a trace of insanity in her family; one of her elder sisters, the mother of several children, and,

* Vide Mistakes of a Night. † Vide Charles O'Malley.

as a mother, entirely free from aught like romantic feelings, was liable to temporary insanity. This lady eventually died of pulmonary consumption.

If there be danger in excitable subjects, unnatural self-control is not, as we have said, without its dangers. The suicide of the late Marquis of Londonderry, better known as the imperturbable Lord Castlereagh of the Lower House, because he conceived himself, in a season of mental exhaustion, slighted by his ministerial friends, affords a memorable instance in high life. In humble life, too, we have seen the most melancholy results from undue suppression of the feelings. The tearless eye but too often denotes a burning brain, and is the harbinger of deadly evils.

Case 4.—While the head-quarters of H. M. 65th regiment lay at Mullinger, in the summer of 1826, the second mess waiter, a very steady exemplary young man, became attached to an equally well-conducted girl, employed as assistant cook in the officers' kitchen. But as the corps was likely, in two or three years, to be ordered on foreign service, the Lieut.-Colonel commanding, from humane motives, wished to discourage marriages among the soldiers beyond the number of women allowed to embark with their husbands when leaving the country—about six women to every hundred men—and, on this account, the young woman was discharged. The attachment, however, continued; and, some time after, the poor creature walked upwards of twenty miles, as well as we recollect, under a burning sun, to implore Colonel D.'s consent to the union. The strict, calculating soldier was, however, inexorable, and the lovers were compelled to take a sad and hopeless farewell. Soon after, we were horrified at seeing some field labourers bringing the girl's corpse, which they had dragged out of the Dublin canal, to the regimental hospital; but although prompt attendance was given, all means were of no avail. The survivor manifested no emotion, but seemed rather to evince revolting indifference. He insisted on attending at the dinner table as usual, and even waited on a supper party—and we need not say an officers' supper is rather a merry one. Early next morning he passed the sentry at the barrack-gate without exciting any suspicion; but was, ere long, brought back, apparently drowned, from the canal, by, we believe, the same men who had seen his sweetheart take the fatal plunge. As his body lay on a table beside that on which hers was stretched, in the *dead-room*, the most gay and thoughtless shuddered at the sight. However, after many efforts, he was restored to life, and placed, carefully watched, in one of the wards; but he persisted in expressing his determination not to survive. The senior medical officer, the late Mr. W. O'Reilly, a man of great skill and judgment, saw the necessity of calling in moral aid; and although an Episcopalian himself, as the Rector of Mullinger was then an aged and infirm man, almost in his second childhood, and his curate too youthful to inspire confidence, called in the Rev. Mr. Gibson, a discreet, middle aged presbyterian minister, much and deservedly respected in the place; and, we are happy to add, his counsels had the desired effect. As teachers of religion, competent to administer to a mind diseased, are not always at hand, and as, on foreign service, in colonial settlements, &c., no minister of religion of any denomination may be near, how desirable is it, however valuable *division of labour* under ordinary circumstances, that the physician should be able to administer, if needful, spiritual medicine. We have sometimes ourselves been compelled to prevent imprudent marriages among soldiers. We have seen, in the discharge of this painful duty, good feeling and refinement that would have done honour to the inmates of a palace; and we have always sought to be kind and considerate; sometimes, in Ireland, where the forlorn damsel was generally a Roman

Catholic, finding a good-natured priest of the old school a very valuable auxiliary in preventing unpleasant consequences.

Case 5.—It is often difficult to draw a just line of demarcation between moral insanity and crime. Revenge, especially long meditated revenge, is confessedly most sinful, and less excusable than hasty resentment. But if, under great provocation, there be apparent apathy, let the friends of the offender and offended beware ; for, granting that the injured party is first stunned, and incapable of feeling resentment at the moment, long afterwards violent reaction may take place. The passionate man is dangerous while his passion lasts, but seldom longer,—as the general rule is, that all violent emotions are speedily exhausted. There is great meaning in Lord Byron's words—

"Cold as cherished hate."

In the year 1815, Mrs. S., a pretty, interesting-looking young woman, wife of a highly respectable butcher, residing in Pimlico, eloped with a neighbouring tradesman, taking with her, as well as we remember, a considerable sum of money. This conduct was the more inexcusable, as the object of her guilty preference was a married, middle-aged man, the father of a numerous family, bloated, sensual, and unintellectual in appearance, and altogether a disgusting profligate; as it afterwards appeared, he had seduced his own wife's sister. On the contrary, Mr. S. was a fond husband, and an intelligent, pleasing man, under thirty; in his holiday clothes looking more like a gentleman than many who claim that title. The morning after this afflicting event, Mr. S. appeared in his shop without change of manner, and whistled to himself, and served his customers, as if nothing extraordinary had occurred. We were then but in our schoolboy days, yet we predicted that, if ever in his power, the injured man would take revenge, and our prediction was verified by the event.

The guilty couple fled to the United States, where Mrs. S. was soon abandoned to poverty by her heartless paramour. At the expiration of two years she contrived to make her way to London in abject misery, and, immediately on her arrival, wrote a most penitent letter to her injured husband, pathetically describing her privations and remorse. He returned no written answer; but, within twenty-four hours, called at her wretched lodging, and without uttering reproach, as she implored his forgiveness, stabbed her three times with a butcher's knife. Providentially, the wounds were not mortal; and, as the offender made no attempt to escape, but rather wished for a legal death, his provocation and general character being taken into account, and his deportment in the dock exciting general compassion, he was sentenced to two years' imprisonment. While undergoing his sentence, his excellent conduct soon led to his being employed as a clerk, or something of the sort, in the prison. We repeat, we do not plead for revenge, however great the provocation; but if we witness, on the provocation being first given, undue apathy, we would advise spiritual counsel to be administered as soon as possible, while the aid of the physician may be not wholly needless to repair the shock of the nervous system. "The waveless calm, the slumber of the dead," is not resignation.

Case 6.—We often have just cause to feel astonished at the imprudence of parents, who are considered, by their neighbours, impersonations of prudence, because they look well to *the main chance*, possess a large, well-furnished house, and "fare sumptuously every day." Such are, in general, the least likely to exercise a well-regulated influence over the moral sentiments of their children, as they progress from the first access of puberty to the flower of their youth. The kind, obedient son, or the gentle, affectionate daughter, is looked on as a mere automaton,—without individuality of volition or feeling; and needful as it is for parents to prevent rash marriages, it is equally needful they should not sacrifice their offspring at the shrine of Mammon, whose worship is quite as unscriptural, and far more degrading, than the adoration of angels and saints, or even of saintly relics.

Towards the close of February last, a benevolent citizen of Paris, M. Laville, an inhabitant of that quarter, met, in the Rue des Martyrs, a young lady, elegantly attired, and of interesting appearance, wandering alone, whose countenance and manners too plainly indicated mental alienation. Having kindly withdrawn her from the inquisitive gaze of a crowd of young people, he conducted her to his own house, and gave her into charge of his wife, who lavished on her the most assiduous attentions; but all the tact of Madame Laville failed to elicit from the interesting stranger any disclosure of her name, or clue to her family. She, however, though obstinately silent on other points, requested the use of a desk and writing materials; and then committed to paper verses, whose impassioned tone too plainly indicated blighted affections to be the cause of her malady.

A few days later, M. Laville addressed a letter to the editor of the *Constitutionnel,* which had already given some account of the fair wanderer, reporting her death. Once only, during seventy-two hours, had she been prevailed on to partake of food. With that exception, her obstinate resistance had baffled all the efforts of her medical attendants to administer nourishment; and, after two hours' intense suffering from frightful convulsions, she rapidly sank under exhaustion, while pronouncing the oft-repeated name of "Robert." We may here pause to inquire whether the determination of the insane to abstain from food always denotes the presence of suicidal mania, or whether it may not proceed from an irritation of the stomach, sympathizing with an excited brain, which causes a loathing of aliment, not less powerful than the horror of water in hydrophobia. Deglutition may also be extremely difficult, from some nervous affection of the œsophagus.

It appears that this ill-fated young lady was the daughter of a wealthy iron-master of Belgium, who wished to unite her in marriage with the son of an equally wealthy brass-founder, of Louvain. Her affections, however, were already bestowed on Robert D—, an artist and author; but alas, like most men of his peculiar talents, little favoured with this world's wealth. She herself was highly gifted and accomplished; and wrote verse with great elegance and facility. On hearing her father's decision, too well knowing his inflexible temper to hope for any change, she sought her lover, and proposed a double suicide. To this he objected; but, the same evening, sought a café frequented by his rival, with whom he contrived to quarrel. Angry words were exchanged, which led to blows, and a challenge ensued. The young men met; and poor Robert D— fell, stabbed through the heart by the sword of his antagonist.

We must in charity hope, if her parents heard of this sad affair, some precautions were taken; but certainly not with due efficacy, as next morning Emilie B— disappeared from home; (in addition to details of her appearance, the initials, E. B., on her linen, were the only clue M. Laville could give towards identification,) and all efforts of her friends to discover the fair fugitive were ineffectual, until her sorrowing father chanced to see the letter to which we have adverted. He instantly repaired to Paris, sought the dwelling of his beloved daughter's last earthly benefactors, and felt but too certain that the deceased maniac was the lost child for whom he mourned. It is needless to say, he manifested the most profound grief; with streaming eyes, he uttered heartrending self-reproaches; and, after showing M. Laville a most touching letter which Emilie had left behind, with some verses of deep pathos, and refusing, with thanks, the proffered hospitality of his kind host, immediately returned to Belgium, although seriously indisposed from grief and fatigue. The days of romance, even in this so-called iron age, are by no means passed away; and, some sixty years hence, perhaps, this tale may afford subject for a fictitious narrative of thrilling interest, entitled, "The Victim of Love; or, The Martyr of Mammon."

We do not, however, mean to speak harshly of M. B——. We only point out his apparent want of judgment; for he plainly was a fond father, and could feel deeply when *his own sensibilities* were touched. Such a young couple as Emilie B—— and Robert D—— are too frequently very unfit to make their way in a world of such cold realities. Persons of their temperament seldom know the value of money; and while there is danger of such fervid affection being soon exhausted by its own ardour, there is also risk of the old adage being realized—"When poverty enters the door, love flies out at the window." The children of a married pair so constituted, would, in all probability be, in some instances, either imbecile, or liable to acute nervous diseases—or wayward and intractable—unless brought up with great care. When there is the poetic temperament on one side, there should be a great portion of "good sense and knowledge of mankind," and a well-developed muscular system on the other, with some dash of the lymphatic temperament: but, at the same time, sufficient taste to appreciate genius, and much kind forbearance towards its too frequent infirmities. Parents should beware how they allow enthusiastic young people to meet, which they often permit without the least apprehension of evil consequences. If affections, through any such negligence, become *mutually* vehement, the most prudent step is, perhaps, to allow matters to take their course, on the principle of preferring the minor evil. Hygienic education may do much to render the children of excitable parents healthy and useful members of society; and the kindly influence of judicious friends may preserve the parents themselves from many evils, until they have learned a little experience and discretion. It is as hopeless to chain the winds, or tame the waves with the lash, as to subdue the impetuosity of love-stricken enthusiasts by coercion. We know that the *Psychological Journal* is perused by many general readers, and for such the greater part of this article is written, (as far as possible in untechnical language), as much as for members of the profession. In cases like the last three which we have detailed, the priest and the physician are too commonly summoned when their aid is too late; and when all must confess the truth of Milton's words,—

"The past who can recal, or done undo?"

As there is but a step from the sublime to the ridiculous, a sudden transition from absurdity to romance is equally possible; but the romance of a madman is a serious affair, as the following statements, which appeared in various Parisian journals early in the month of May, this year, will testify.

Case 7.—The Sieur D——, carrying on business in the Quartre St. Denis, had, for some time, given unmistakable evidences of mental aberration. He frequently sent goods to parties without any order; often went out without his hat (unlike a certain Englishman who used to insist, when deranged, on wearing two); and repeatedly returned laden with children's toys and useless purchases; sometimes treating his subordinates with great rudeness, and charging his head clerk, a man above all suspicion, with dishonesty.

Madame D——, well skilled herself in the transactions of business, palliated, to the utmost of her power, his eccentricities and offensive conduct, which had not, as yet, become at all dangerous; and by earnest and respectful attentions induced the assistants to bear with the caprices of their employer; exerting all her ingenuity, day after day, to conceal from their customers the sad reality.

On, or about the 3rd of May, Madame D—— was awoke, an hour after midnight, by a painful sensation in the throat; and on raising her hand to discover the cause, ascertained, with terror, that her husband, who stood before her, was attempting to strangle her with a cord, of which he held one end, while he regarded her with a strange wild look. At first she thought this horrible reality only an hallucination, and rubbed her eyes to assure herself she was not deceived. On the table lay an open razor and a pistol.

"Come, my best beloved," said the husband, with a smile which made his countenance still more frightful, "it is time to set out on our journey. They expect us, for our nuptials are to be celebrated on high, in the moon. We shall leave our bodies here—they will give us others; and we shall resume our own after a few days. Come, take away thy hands, that I may strangle thee, to cause thy spirit to depart! Seest thou, on high, all the invited guests who pass?" At the same time he directed his wife's attention through the window, the curtains of which had been drawn aside, to some light clouds flitting across the disc of the moon.

At this critical moment a happy thought occurred, as if by inspiration, to Madame D——. Appearing to enter into the maniac's feelings, she calmly answered, "I cannot, dearest, consent to go on high before thee; and if we leave our bodies here, without explanation, they will be buried in the cemetery; or rather, it may be, opened for examination." "Thou art right," was the answer; "I did not think of that. I will just go and write a couple of lines: we have no time to lose; and thou must go first to put on thy apparel."

Providentially there was no ink in the room; and the Sieur D—— was therefore compelled to descend to his office on the ground floor. Soon as he had left the apartment, Madame D—— gently closed and bolted the door. She then opened the casement, which looked into an inner court, and threw, one by one, several pieces of money at the opposite windows. As she had foreseen, one of these casements was soon opened by a neighbour, to whom she had confided the state of her husband, and to whom she now hastily communicated her danger, requesting him to call the nearest guard to her aid. The soldiers were promptly on the spot; and forcing their way into the house, found the Sieur D—— proceeding to break open the bed-room door with a wrenching iron. It was necessary to use stratagem to gain possession of this formidable weapon and secure his person, as he had become exceedingly violent. His arms and legs were then firmly tied, and, in this state, he was conveyed to the guard house. Next day he was taken to a lunatic asylum, where he was immediately secured with the *strait waistcoat.*

Here we would observe, when the padded-room will not suffice, a very loose dress, of some very strong material, tied under the feet, with the sleeves also tied below the hands, may be usefully employed to prevent mischief, the patient being either placed on a wide bed, so secured with padded head, foot, and side boards, as to prevent his rolling out, or laid on the floor of a padded-room, well littered with hay or straw. The more he is at liberty, with such precautions, to kick, sprawl, and vociferate, the sooner his fury will probably sink into the tranquillity of exhaustion.

Whether the effects of the bite of the tarantula were produced by the poison of the insect, or by fear acting on the imagination, dancing, by carrying off the excess of the nervous excitement, was found the best remedy; the music, specimens of which may be found in "Hecker's History of the Epidemics of the Middle Ages," being rather solemn and soothing. Some physicians, supposing the virus of the poison carried off by cutaneous transpiration, tried diaphoretics, but without success, as appears from Baglior's Writings, among others. Sudorifics neither exercised the limbs nor amused the mind.

We have often been surprised, considering the number of insane and (Scotticè) "fatuous persons," always at large in Ireland, or only under the very inefficient control of their friends (even now the number of such is estimated at 6000), that so few unpleasant consequences ensue from this obvious want of precaution. Among the middle and higher classes, the old "fire eater" (or professed duellist) has happily disappeared with the progress of civilization; gentlemen now substituting the arrows of the tongue and the gray goose shaft, for sword and pistol—preferring the platform to *the sod*, and the steel-pen to *cold iron*. But the Irish peasant, with all his good qualities, and they are many, is still what his ancestors were centuries ago, a very dangerous character when excited by anger, revenge, or whisky; and yet, when deranged, he is, by what he would himself call "the rule of contrary," very often a most harmless character, and sometimes highly amusing. This, in a great measure, may be owing to the kindness of the more humble classes; who, like the people of the east, consider the imbecile and the insane especial favourites of heaven; and so bear, with incredible patience, the follies and extravagancies of *the poor innocents*, who are looked upon, especially if imbecile from birth, with peculiar interest, as incapable of committing *actual sin*. Having so far entered into so many melancholy details of insanity, we may, we trust, in conclusion, give one instance of its ludicrous manifestation in an Irish lunatic, from the statements of an elder friend, now no more, who witnessed his vagaries.

Some forty years since, a poor discharged soldier, a fine looking man, in the prime of life, and of very military and dignified demeanour, who, we believe, had become insane in the West Indies from the effects of a severe flogging,—a punishment wholly unsuited to men of high spirit—appeared in the town of Mallow one day, attired in the cast-off uniform and cocked hat of an officer; his head-gear adorned with a plume of peacocks' feathers, and a ponderous wooden sword, in a leathern scabbard, dangling by his side. As this hero stalked through the streets, he gravely announced himself—"Colonel Jack, commander in chief of all the milk women in Ireland."

Next morning Colonel Jack visited the milk market at an early hour, marshalling its nymphs, between twenty and thirty in number, seated in line on their stools, their tin milk-pails, with the lids closed before them, and their measures duly arranged. Then standing in front of the amazons—for such were some, at least if provoked—he gave the word of command in a loud voice—*Open pans;* on which, in accordance with their previous instructions, all the lids were raised. The gallant colonel forthwith minutely examined, not only the pails and measures, but the faces, hands, and dress of each lady fair; kicking over her milk without ceremony, if there appeared to be any want of attention to cleanliness. The women laughed, and endured their misfortunes with the utmost good humour; there were no "peelers" at the time to interfere; and the magistrates and town constables, when they heard of the matter, thought it not only a good but a useful joke. Daily, for six months, Colonel Jack attended morning parade; but, after the first significant demonstration of his antipathy to dirt, had little or no cause to inflict punishment. At the end of half a year, he disappeared as abruptly as he had made his appearance—no doubt on an extended *tour of inspection;* but what afterwards became of him we could never learn.

Once, and once only, did *the Colonel* deviate from his established routine of duty, as well as we remember, by entering a school, where, politely bowing to the master, as he asked the loan of his cane, he commenced inspection of faces and hands among the pupils, using the *rod of office* without hesitation, if ablution had been neglected. "The dominie," all this time tried to laugh, and appear amused; but when the boys' pains and penalties were over, Jack insisted that the pedagogue himself should show his palms; and as they were not up to the mark, to the infinite delight of his urchin subjects, he was compelled to hold out for a couple of *pandies;* after which, the inspector, replacing the rattan on the desk, courteously withdrew—leaving a recollection of his visit which, although never repeated, led to a very increased consumption of soap and water in the establishment while he continued to sojourn in the good town of Mallow.

May we not take a hint, from a case like this, of, far as practicable, humouring the insane in their harmless fancies, until they are sufficiently brought under curative treatment to arouse them from their delusions. Colonel Jack, a Saxon would reasonably think, was allowed to go too far; and certainly, had he met any annoyance in either the market or school, he would probably have proved "a rough customer;" but really on such occasions, the patience of the Irish used to be inexhaustible; and the same benevolent feeling has rendered them most kind nurses of children and of the infirm and the aged. For persons of unsound mind, Ireland, in our early days, was a veritable "paradise of fools." We can call to mind one gentleman, possessing about £2000 a-year, in the Cove of Cork, who thought he had lost his fortune, and was allowed to wander about the country, selling needles and thread, penknives and scissors, &c.; or, at least, supposing he sold them. We have also seen in England, not long after the Battle of Waterloo, a captain placed on half-pay, in consequence of insanity, who resided at Chelsea, permitted to amuse himself by supposing he cheated the keepers of stalls in the street: he would call for, according to the season, fruit, or oysters, and after eating them, walk off in triumph when asked for payment, without noticing the demand. However, as he never incurred debt beyond a few pence, his keeper, who followed a little behind, always settled the accounts; and, consequently the captain uniformly met a very gracious reception.

It will not, however, answer any good purpose to allow our benevolence to interfere with proper precautions. Even in Ireland, melancholy exemplifications of the evil results of negligence occur. We have heard, for instance, of a young lady, residing in a rural district, on the eve of the day appointed for her wedding, going to the shop of a neighbouring village to purchase her bridal ribands, and such like adornments; and while, in all the happiness of hope, returning homeward, being assaulted and violated, as she crossed a lonely churchyard, by a hideous idiot, who, up to that period, had never been known to inflict serious injury on any one. As a general rule, scarcely admitting of exception, however apparently inoffensive,—THE INSANE AND THE IMBECILE SHOULD NEVER BE UNWATCHED, unless with the sanction of some eminent practitioner in mental pathology, when about to be released from restraint, or where a sense of being watched might only aggravate their malady in its incipient or very mild stages. To judge of the cases in which this liberty can be allowed, even for a few hours in the day, requires not only long experience, but great natural power of discrimination. There is, thank God, much benevolent feeling in the present day; but, as extremes meet, injudicious mildness may be as fatal as wanton cruelty.

